# Supplementary material for: Chromothripsis during telomere crisis is independent of NHEJ, and consistent with a replicative origin
Source: Genome Res. 2019 May;29(5):737–49. doi: 10.1101/gr.240705.118 (PMC6499312; doi:10.1101/gr.240705.118)
Supplement: Supplemental Material [file supp_gr.240705.118_Supplemental_file_1.zip › contigs/annotated_contigs/DB37/contig.2.DB37_length_560_mean_cov_3.41964285714.docx]

**DB37_length_560_mean_cov_3.41964285714**

```
CTTGATGCTCTAAGTTCTTGATGCCTGTGTGATTTCTTATTTTGATGACATTTTCCAATGCCACGTGATATTTTTGAAATGAAAAAATT
 >chr18:75012321-75012769 + E=3e-258
TGTACATTTTGATCAACTTGCCAGCAAACACCCCTTGAGAGCCCTCCAGCTCCAGGAGCTTAGACGTCGTGACTCCGGCACCCACCGGA

TGCTCCACTCACTTTGTTTTGGCCTTCACACAACAAACCTGACTTTAATACAAACCCAACTGAAAAGCACTGGACGTTGCAAGACTTCA

AAGGCCACTCATCAGCACCTTTCCCAAGCTCCTGCTTGCCACCTGCACATGACAGATGTGACAGCTTAGACGAAACCCATGAGGTTTTT

CTCCCCAGCCTTTGGTGCGGAGCTCCCATCTGGAGCCGTGTTTCATCTGGCACTGCGGGGCATGAGAACTAACAGCTGGTTAATGGCC|

TGGC|ACTACTACTGTTTTTGAGATCATTTAATCAGTAAAAGAGAAGGGTGCCATAGCAAAATGCCTTATGTTGGCATCTGCATGAAAT
>chr18:75014004-75014120 - E=4e-58
CTCTTCTTACAACAGTGCCTAGTTTTCT
```
